# A Review of Plasma-Synthesized and Plasma Surface-Modified Piezoelectric Polymer Films for Nanogenerators and Sensors

**DOI:** 10.3390/polym16111548

**Published:** 2024-05-30

**Authors:** Eun-Young Jung, Habeeb Olaitan Suleiman, Heung-Sik Tae, Choon-Sang Park

**Affiliations:** 1 The Institute of Electronic Technology, College of IT Engineering, Kyungpook National University, Daegu 41566, Republic of Korea; eyjung@knu.ac.kr; 2 School of Electronic and Electrical Engineering, College of IT Engineering, Kyungpook National University, Daegu 41566, Republic of Korea; suleiman.habeeb16@knu.ac.kr; 3 Electrical Engineering, Milligan University, Johnson City, TN 37682, USA

**Keywords:** flexible nanogenerators, piezoelectric polymer film, plasma polymerization, plasma surface modification

## Abstract

In this review, we introduce recently developed plasma-based approaches for depositing and treating piezoelectric nanoparticles (NPs) and piezoelectric polymer films for nanogenerator (NG) and sensor applications. We also present the properties and an overview of recently synthesized or modified piezoelectric materials on piezoelectric polymers to highlight the existing challenges and future directions of plasma methods under vacuum, low pressure, and ambient air conditions. The various plasma processes involved in piezoelectric NGs and sensors, including plasma-based vapor deposition, dielectric barrier discharge, and surface modification, are introduced and summarized for controlling various surface properties (etching, roughening, crosslinking, functionalization, and crystallinity).

## 1. Introduction

Energy harvesting is currently receiving significant attention [1,2,3,4,5]. Recently, the necessity for sustainable and miniaturized power sources has increased due to the increasing demand for portable and wearable electronic devices. However, the ability to satisfy this increasing need for wearable electronics is largely dependent on the availability of suitable power sources. Accordingly, it is important to develop sustainable and renewable nanogenerators (NGs) for energy harvesting [6,7]. These NGs, especially piezoelectric NGs (PENGs) and triboelectric NGs (TENGs), are promising candidates for self-powered electronics [8,9,10,11,12,13]. These PENGs and TENGs change mechanical energy derived from human activities (such as pressure, bending, and stretching motions) into electrical energy [6,7,11,12]. TENGs generate electricity through frictional charges between two materials through electrostatic induction and triboelectrification [11]. In contrast, PENGs generate electric signals by rotating electric dipoles through the deformation of piezoelectric materials [13,14]. Thus, to realize flexible NG devices, numerous researchers have investigated piezoelectric ceramics, including zinc oxide (ZnO), lead zirconate titanate (PZT), barium titanate (BaTiO_3_), and lead magnesium niobate–lead titanate (PMN–PT) [15,16,17,18,19]. However, these piezoelectric ceramics have some constraints when used for flexible NG applications [17]. In addition to piezoelectric ceramics, piezoelectric polymers have attracted attention in the field of flexible PENGs. In particular, polyvinylidene fluoride (PVDF) and poly(vinylidenefluoride–co–trifluoroethylene) (P[VDF–TrFE]) copolymers have been researched due to specific properties, including their mechanical flexibility, piezoelectricity, dielectric properties, high chemical resistance, and good thermal stability. However, the dielectric constant and piezoelectricity of piezoelectric polymers are relatively low compared to piezoelectric ceramics [11,18,19]. Therefore, the development of piezoelectric polymers with a high dielectric constant is necessary to industrialize their application. Many studies have been directed toward developing piezoelectric polymers, including polyacrylonitrile (PAN), PVDF-based copolymers, and polymer nanocomposites, such as piezoelectric nanoparticles (NPs) [13,20,21,22,23,24,25,26,27,28]. These piezoelectric polymers are mainly prepared using wet-based conventional techniques, such as inkjet printing, screen printing, electrospinning, and spin–coating [29,30]. However, these methods are unsuitable for flexible NGs because they are complex, dangerous, and thermal processes. To solve this problem, low and atmospheric pressure plasma (APP) processes have recently attracted attention because of their specific properties, such as pinhole-free and cross-linked structures for polymer film deposition and plasma surface modification [31,32,33,34]. Recent research related to plasma processes for piezoelectric NPs and polymers has also been conducted [35,36,37,38,39,40,41]. Here, we will deal with polymerization and applications using plasma in more detail. 

In this review, we discuss plasma synthesis and plasma surface modification of piezoelectric polymers for NGs and sensor applications. First, the relevant literature on piezoelectric ceramic NPs and polymer film deposition by plasma is examined and discussed, followed by a review of plasma surface modifications. The main purpose of this review is to provide a reference on recent plasma processes for piezoelectric polymerization and plasma surface modification, while briefly discussing the characteristics of piezoelectric polymers prepared using plasma processes. 

## 2. Plasma Process

### 2.1. Plasma Deposition and Synthesis Process of the Piezoelectric ZnO NPs and Polymers

Before discussing plasma polymerization, we will first briefly introduce the piezoelectric ZnO film deposited by using plasma deposition and then discuss plasma polymerization.

#### 2.1.1. Plasma Deposition and Synthesis Process of the Piezoelectric ZnO NPs

**Figure 1:**
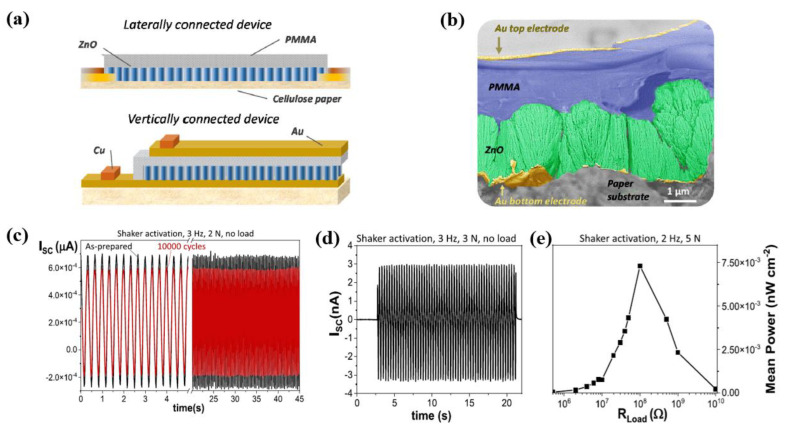
(**a**) Schematic diagram of PENGs devices, by García-Casas et al. (**b**) Cross-sectional SEM image of a multilayer structure with a paper/Au/ZnO/PMMA/Au. (**c**) Output current signals of PENG device before and after long-term operation of more than 10,000 cycles. (**d**) Output current acquired by using magnetic shaker tapping actuation under a constant force of 3 N and fixed frequency of 3 Hz. (**e**) Mean power density according to load resistance values at 2 Hz and 5 N. Reproduced with permission from ref. [35].

García-Casas et al. [35] investigated the piezoelectric nano-sensors and PENGs based on ZnO films (up to 6 μm) fabricated by a plasma-enhanced chemical vapor deposition (PECVD) on commercially available paper substrates. As depicted in Figure 1a, the PENGs devices were assembled by embedding the ZnO films in polymethylmethacrylate (PMMA) and using Au thin layers as electrodes in two different configurations: laterally and vertically contacted devices. A cross-sectional image of the multilayer structure with a paper/Au/ZnO/PMMA/Au was obtained using a scanning electron microscope (SEM), as depicted in Figure 1b. After long-term operation for more than 10,000 cycles, the electrical performance of the PENG device was studied. As displayed in Figure 1c, when the PENG device continued operating for 10,000 cycles, the resulting output current decreased slightly by less than 15%. As depicted in Figure 1d, the performance of the plasma-produced devices exhibited high repeatability of the outcome signal and fast response through actuation with a magnetic shaker for a fixed force and frequency (up to 10 Hz). The mean power density according to the load resistance of the PENGs device is presented in Figure 1e, with a maximum value at an impedance of 108 Ω. Thus, the PENGs device exhibited an instantaneous power density of 80 nW/cm^2^ with a mean power output of 20 nW/cm^2^ [40].

**Figure 2:**
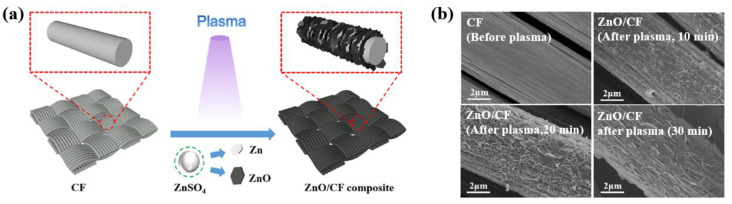
(**a**) Experimental procedure of ZnO/CF composite by plasma–liquid process, by Zhong et al. (**b**) SEM images of ZnO/CF composite before and after the plasma process at various times (10, 20, and 30 min). Reproduced with permission from ref. [36].

Zhong et al. [36] investigated the piezoelectric response properties of ZnO/carbon fiber (ZnO/CF) composites produced by plasma–liquid interaction. To produce the ZnO/CF composites, firstly, the mixed ZnSO_4_ solution was prepared by mixing ZnSO_4_·7H_2_O with ultrapure pure water. Then, the washed fabric was immersed in the mixture and connected to the negative electrode of a power supply. A stainless-steel needle was then connected to the positive pole of the power supply. The power supply voltage was 1.5 kV, and the ZnO/CF composite was synthesized by plasma discharge generated by a liquid plasma system, as depicted in Figure 2a [36]. From the SEM images in Figure 2b, it is evident that the ZnO film was grown on the CF surface with a lamellar nanostructure structure with different plasma process times. 

**Figure 3:**
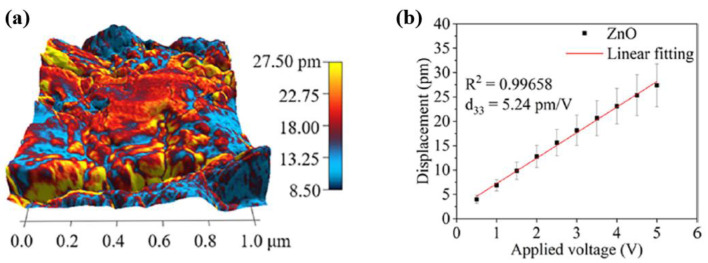
(**a**) Surface amplitude distribution obtained by a piezoresponse force microscopy (PFM) and (**b**) linear fitting plot of amplitude with an applied voltage in the ZnO/CF composite, by Zhong et al. Reproduced with permission from ref. [36].

To evaluate the piezoelectric response property of the ZnO layer on the CF surface, the surface charge distribution and piezoelectric coefficient (d_33_) values of the ZnO/CF composite were acquired using piezoresponse force microscopy (PFM). As depicted in Figure 3a, the amplitude distribution of the ZnO surface was observed under an applied AC voltage, which confirmed the piezoelectric response of the ZnO/CF composite. Moreover, as demonstrated in Figure 3b, the displacement response increased linearly with the applied voltage amplitude. Based on the linear fit of the amplitude of the displacement with an applied voltage, the d_33_ of the ZnO/CF composite was obtained, with a value of 5.24 pm/V [36]. 

**Figure 4:**
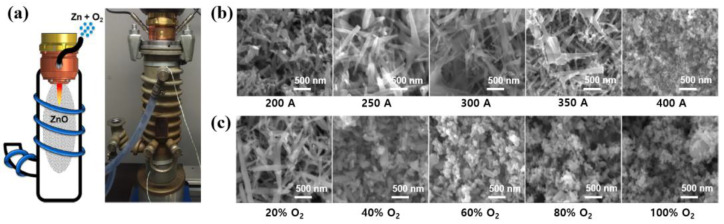
(**a**) Scheme and photo image of plasma jet reactor for ZnO NP synthesis, by Schwan et al. SEM images of the synthesized ZnO NPs with different (**b**) discharge current and (**c**) oxygen flow rate conditions. Reproduced with permission from ref. [37].

Schwan et al. [37] examined an atmospheric pressure plasma jet (APPJ) reactor for ZnO NPs synthesis by using ZnO powder and oxygen gas. Here, ZnO NPs were synthesized from zinc powder and oxygen gas in the APPJ reactor chamber using plasma flight-thru synthesis, as depicted in Figure 4a. The commercial APPJ reactor (IC3, INOCON Technologie GmbH, Attnang-Puchheim Österreich, Austria) was operated with argon as the plasma gas (10 L/min) and mixtures of argon and oxygen as the powder carrier gas. The zinc powder was provided through powder feeding injection. To synthesize the ZnO NPs using a plasma flight-thru technique, a direct current thermal plasma reactor was operated at atmospheric pressure. The injected ZnO powder was melted, vaporized, and oxidized by the plasma process to facilitate ZnO NPs growth [37]. Based on the SEM results in Figure 4b,c, it was confirmed that the synthesized ZnO NPs could be controlled in terms of shape and size through the discharge parameters (oxygen gas flow and plasma discharge current) [37]. 

**Figure 5:**
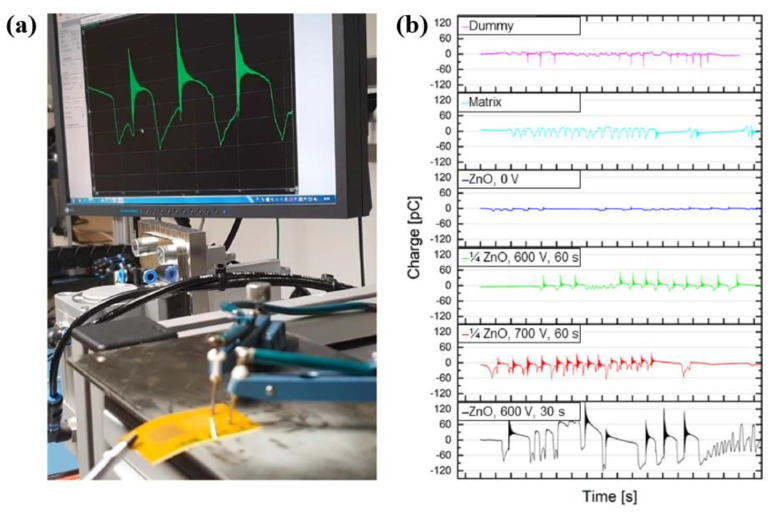
(**a**) Experimental setup for the piezoelectric measurement, by Schwan et al. (**b**) Piezoelectric charge properties obtained by piezoelectric test sensors with different ZnO NPs. Reproduced with permission from ref. [37].

Furthermore, the piezoelectric properties were also measured using piezoelectric test sensors with different ZnO NPs, as depicted in Figure 5a. The piezoelectric signals were detected according to the mechanical stimulus when the ZnO NPs were dispersed in a matrix of acrylic resin and fixed between finger-electrodes while poling, as displayed in Figure 5b [37].

**Figure 6:**
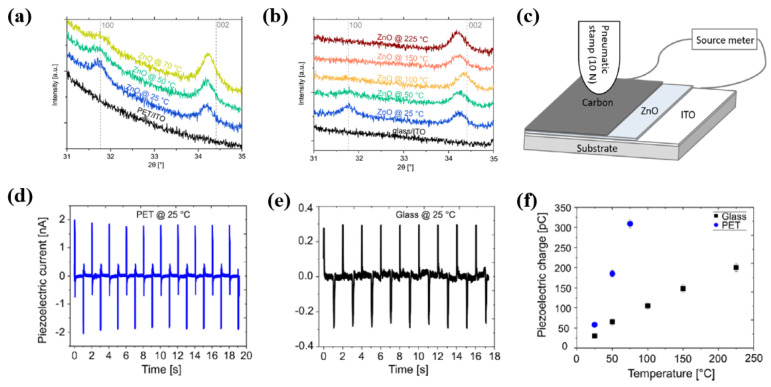
XRD patterns of ZnO thin films grown on (**a**) PET and (**b**) glass with different substrate temperatures. (**c**) Schematic diagram for device structure and piezoelectric measurements. Piezoelectric current signals of ZnO thin films grown on the (**d**) PET and (**e**) glass. (**f**) Piezoelectric charge properties of ZnO thin films grown on glass and PET, by Ali et al. Reproduced with permission from ref. [38].

Ali et al. [38] examined ZnO thin films prepared by plasma-enhanced atomic layer deposition (PE–ALD). Using the PE–ALD technique, the ZnO thin films were grown with different substrate temperatures to investigate the effect on crystalline and piezoelectric properties. From the XRD results displayed in Figure 6a,b, it is evident that the crystallinities along the (002) plains increased with increasing substrate temperature, which could be related to enhanced piezoelectric output. The piezoelectric properties were also measured in the piezoelectric test device with ZnO thin films, as depicted in Figure 6c. As a result, the ZnO films grown on flexible poly(ethylene terephthalate) (PET) substrates exhibited a higher piezoelectric current compared to the rigid glass substrate because of mechanical bending effects, as depicted in Figure 6d,e. This current enhancement was attributed to increased piezoelectric charge caused by the mechanical bending, which was confirmed by the PFM measurements displayed in Figure 6f [38]. Table 1 summarizes the plasma deposition and synthesis processes for piezoelectric NPs presented in this section.

#### 2.1.2. Plasma Deposition and Synthesis Process of the Piezoelectric Polymer Using APP Technique

**Figure 7:**
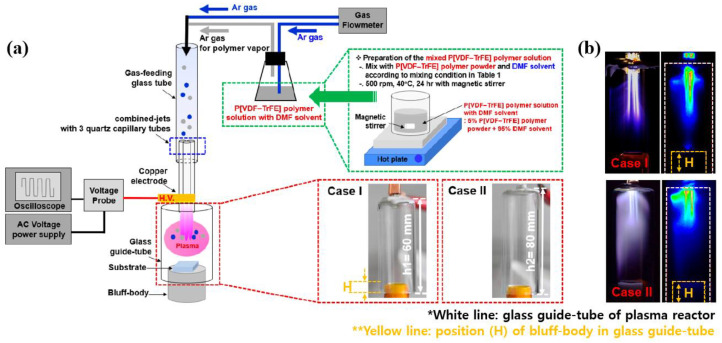
(**a**) Experimental setup of APP system for PVDF and P[VDF–TrFE] thin film deposition, Jung et al. (**b**) Photographs and intensified charge-coupled device (ICCD) images of plasma produced by APP deposition with two different lengths of glass guide-tubes (Cases I and II) [39,40].

Jung et al. [39] examined the structural and dielectric properties of P[VDF–TrFE] copolymer thin films grown by APP deposition using a mixed polymer solution comprising P[VDF–TrFE] nanopowder and dimethylformamide (DMF) solvent, as depicted in Figure 7a. In the APP deposition system, the length of the glass guide-tube is an important parameter when producing intense cloud-like plasma for polymer deposition. As displayed in Figure 7b, a different intensity of cloud-like plasma was observed in the glass guide-tube length of Case II compared to Case I. Ultimately, the P[VDF–TrFE] thin film was uniformly deposited to a thickness of 3 μm. 

**Figure 8:**
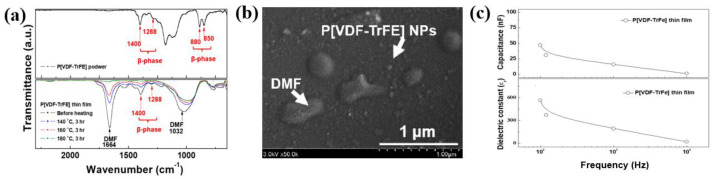
(**a**) FT-IR spectra of P[VDF–TrFE] thin film with various post-heating temperatures. (**b**) SEM image, and (**c**) capacitance and dielectric constant of post-heated P[VDF–TrFE] thin film prepared by APP deposition after post-heating at 160 °C for 3 h [39].

P[VDF–TrFE] thin films with excellent β–phase structural properties were deposited using APP deposition under optimum conditions at room temperature for 1 h. However, large amounts of DMF elements remained in the P[VDF–TrFE] film after deposition. Therefore, post-heating treatment was performed on a hotplate in air for 3 h at temperatures of 140, 160, and 180 °C to remove any remaining DMF solvent and obtain pure piezoelectric P[VDF–TrFE] thin films. As a result, the Fourier transform infrared spectroscopy (FT–IR) results in Figure 8a confirm that the peak intensity for DMF decreased with increasing the post-heating temperatures. Thereafter, the post-heated P[VDF–TrFE] thin films exhibited crystalline peaks of β–phases. From the SEM results presented in Figure 8b, it is evident that the post–heated P[VDF–TrFE] thin films at 160 °C had a smooth surface with P[VDF–TrFE] NPs. Moreover, as displayed in Figure 8c, the capacitance and dielectric constant values decreased with increasing frequency due to dipole dispersion in the polymer structures [39]. Thus, the dielectric constant of the post-heated P[VDF–TrFE] thin film at 160 °C for 3 h was 30 at 10 kHz and room temperature. Accordingly, post-heated piezoelectric P[VDF–TrFE] copolymer thin film prepared by APP deposition is expected to be a prospective piezoelectric polymer material for flexible PENG [39]. 

**Figure 9:**
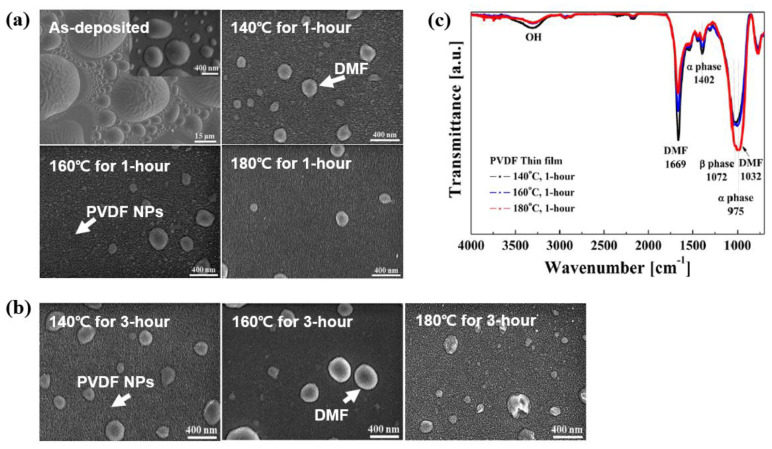
SEM images of PVDF thin film deposited by APP deposition before and after post-heating for (**a**) 1 and (**b**) 3 h with various post-heating temperatures [40]. (**c**) FT-IR of PVDF thin film deposited by APP deposition with various post-heating temperatures for 1 h. Reproduced with permission from ref. [40].

Jung et al. [40] systematically investigated the effects of post-heating treatment on the crystalline phase of a PVDF thin film fabricated through APP deposition using a PVDF/DMF solution. The surface morphology and chemical structural properties were examined with different post-heating temperature conditions (to eliminate the DMF elements and enhance the crystalline phase) using SEM and FT-IR [40]. Figure 9a shows the SEM images of PVDF thin film deposited by APP deposition before and after post-heating for 1 h with various post-heating temperatures at 140, 160, and 180 °C. In case of as-deposited PVDF thin film, the deposited film was observed to have a rough surface covered with bubbles by DMF vapor. Meanwhile, for post-heated PVDF thin film, the amounts of bubble and corresponding bubble sizes were decreased, as the post-heating temperature was increased. It is evident that increasing the post-heating temperature conditions decreased both the amount and size of the bubbles. Furthermore, PVDF NPs were clearly observed on the surface of the PVDF thin film. Additionally, in order to effectively reduce the DMF element and improve the crystalline phase of PVDF thin film, the PVDF thin films were post-heated for 3 h with increasing the post-heating temperatures. Figure 9b shows the SEM images of PVDF thin film deposited by APP deposition before and after post–heating for 3 h with increasing the post–heating temperatures. As post-heating temperatures increases, both the amount of bubble and the size of bubble were slightly decreased in the PVDF thin film from the SEM results in Figure 9b. As a result of the experiment, the changes in post-heating time have no significant effect on improving the change in surface properties of the PVDF film.

From the FT-IR spectra in Figure 9c, as the post-heating temperatures increased from 140 to 180 °C for 1 h, the peak intensity at 1669 cm^−1^ for –C=O by the DMF solution decreased significantly, indicating that the DMF component was effectively removed. Moreover, the crystalline phases of the post-heated PVDF thin film mainly exhibited two phases (α and β). These were indicated by peaks at 975 and 1402 cm^−1^ for the α-phase and a peak at 1072 cm^−1^ for the β-phase [40].

**Figure 10:**
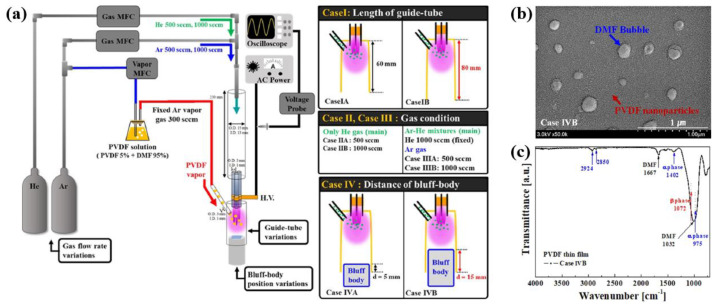
(**a**) Experimental setup of the modified APP deposition system (modified–APPDS) with direct-injection nozzle used in this study for PVDF film deposition, by Bae et al. (**b**) SEM image and (**c**) FT–IR of PVDF film after post-heating at 160 °C. Reproduced with permission from ref. [41].

The conventional APP system tends to lose its monomer precursor before injecting the plasma polymerization region. To minimize this monomer precursor loss, Bae et al. [41] suggested the modified APP deposition system (modified-APPDS) with a direct-injection nozzle for PVDF thin film deposition. As displayed in Figure 10a, the precursor monomer vapor was injected into the plasma reactor separately from the Ar gas flow. In the modified–APPDS process, to obtain the optimal conditions for generating glow-like intense plasma, case studies were examined with various discharge parameters such as the length of the guide–tube, the distance of the bluff-body, gas composition, and gas flow rates. As a result, intense glow–like plasma was produced in the modified–APPDS with optimal conditions (Case IVB), and PVDF thin film was uniformly deposited [41]. The deposited film thickness was measured at approximately 1 μm by using a stylus profiler. After PVDF film deposition, the PVDF thin films were heated on a hotplate at 160 °C for 3 h to eliminate any remaining DMF elements and enhance the crystalline phase [41]. As displayed in Figure 10b, impurities in the form of bubbles were observed in the deposited PVDF film, which was attributed to the DMF solution. Finally, the PVDF NPs were deposited as a uniform film [41]. As depicted in Figure 10c, the PVDF thin film mainly consisted of two crystalline structures of α- and β-phases, which was confirmed by the FT-IR spectra. Table 2 summarizes the plasma deposition and synthesis process of piezoelectric polymers using the APP technique discussed in this section.

### 2.2. Plasma Surface Modification of the Piezoelectric ZnO and Polymers Using Plasma Process

In addition, we will deal with material properties and device performance improvement by plasma surface modification of the piezoelectric ZnO and polymer to improve the electrical performance of sensors and NGs devices.

#### 2.2.1. Plasma Surface Modification of the Piezoelectric ZnO Film for Sensor Application 

**Figure 11:**
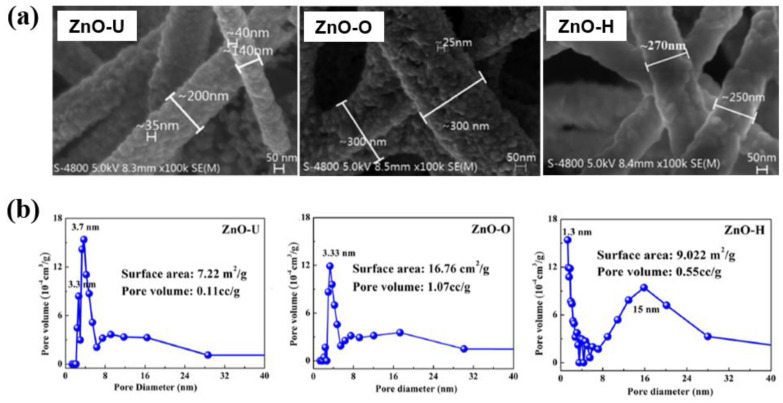
(**a**) SEM images and (**b**) pore distributions of ZnO NFs before and after plasma treatment for 30 min using O_2_ and H_2_ gas conditions, by Du et al. Reproduced with permission from ref. [42].

Du et al. [42] investigated the gas-sensitive response for plasma-treated ZnO nanofibers (NFs). For experiment, ZnO NFs were prepared by electrospinning through spinning solution with zinc nitrate [42]. After that, the electrospun ZnO NFs were treated by radio frequency (RF) plasma using a low power inductively coupled plasma source (ICPS) at an operating frequency of 13.56 MHz. The pressure of the vacuum chamber and the discharge power were 30 Pa and 450 W, respectively [42]. The ZnO NFs were treated for 30 min with two different oxygen (O_2_) of 14 sccm and hydrogen (H_2_) gas conditions. Here, the untreated, O_2_, and H_2_ plasma-treated ZnO NFs were indicated as ZnO-U, ZnO-O, and ZnO-H, respectively. Figure 11a shows the SEM images of ZnO NFs before and after plasma treatment for 30 min using O_2_ and H_2_ gas conditions [42]. For ZnO-U sample, the width of fibers and particle size of ZnO NPs are 200 nm and 35 nm, respectively. In case of ZnO-O sample, the width of fibers increased to 300 nm and the particle size of ZnO NPs on the NFs is smaller compared to the ZnO-U sample. In addition, ZnO-H sample has a smaller width than ZnO-O and a larger width than ZnO-U. The specific surface area and porosity of the ZnO NFs samples were measured by using N_2_ porosimetry before and after plasma treatment for 30 min using O_2_ and H_2_ gas conditions. As shown in Figure 11b, for ZnO-U sample, the specific surface area, pore size, and pore volume were 7.22 m^2^/g, 3.7 nm, and 0.11 cc/g, respectively. In addition, the specific surface area of ZnO-O sample increased to 16.67 m^2^/g, and that of ZnO-H was 9.022 m^2^/g [42]. Thus, the surface morphology of ZnO-O NFs was changed and has more pores with a larger specific surface area due to O_2_ plasma treatment.

**Figure 12:**
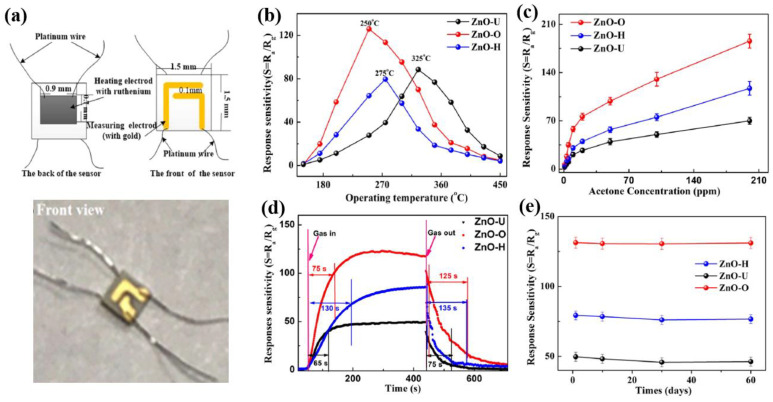
(**a**) Schematic diagram (upper) and photo image (bottom) of an indirect-heated ceramics gas sensor, by Du et al. [42]. (**b**) Response sensitivity of gas sensor with different temperatures at a 100 ppm of acetone according to the different ZnO NFs samples before and after plasma treatment. (**c**) Response sensitivity of sensors with various acetone concentrations at a temperature of 250 °C. (**d**) Response and recovery times of gas sensor at a 100 ppm of acetone according to the different ZnO NFs samples before and after plasma treatment. (**e**) Repeatability of gas sensor for 60 days at 250 °C, in acetone. Reproduced with permission from ref. [42].

To evaluate the sensor performance, an indirect-heated ceramics gas sensor was fabricated as shown in Figure 12a [42]. Figure 12b shows the response sensitivity of gas sensor with different temperatures at a 100 ppm of acetone according to the different ZnO NFs samples before and after plasma treatment. When compared to the ZnO-U sensor, the sensor operating temperatures of ZnO-O and ZnO-H samples were reduced to approximately 75 and 50 °C, respectively [42]. In addition, the response sensitivity of gas sensors with various acetone concentrations in ranges from 1 to 200 ppm at 250 °C for three types of ZnO sensors is shown in Figure 12c. As a result, the ZnO-O sensor shows a high response to a low concentration of acetone, and the ZnO-U sensor has the lowest response. Thus, the response sensitivity of the ZnO–O sensor increases, and the ZnO-H sensor has a higher response sensitivity at 250 °C when compared to ZnO-U sensor. Moreover, the response and recovery times of ZnO sensors were measured and shown in Figure 12d [42]. In Figure 12d, the response and recovery times of ZnO-O sensor are approximately 75 and 125 s, respectively, which are longer than those of ZnO-U with 65 and 75 s, and shorter than those of ZnO-H with 130 and 135 s [42]. Furthermore, the response performance of ZnO sensors was well maintained after the ZnO sensors were operated for 60 days as shown in Figure 12e [42].

**Figure 13:**
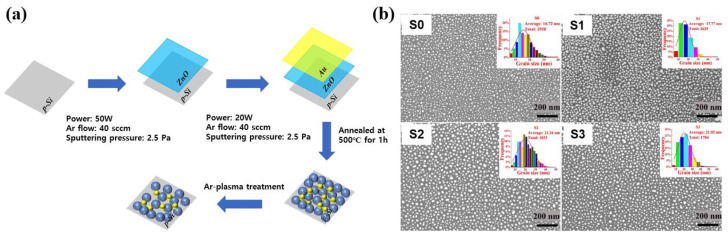
(**a**) Experiment procedure process of Au-ZnO films with Ar plasma treatment, by Wang et al. [43]. (**b**) SEM images of before and after plasma treatment with different treatment time conditions. Reproduced with permission from ref. [43].

Wang et al. [43] investigated the gas sensor response properties according to plasma treatment of Au-ZnO films prepared by combining the magnetron sputtering and the Ar plasma treatment. To produce the Au-ZnO films, firstly, the ZnO films were deposited on the Si substrates by RF magnetron sputtering through ZnO and Au targets under a base pressure of 1.6 × 10^−4^ Pa, operating pressure of 2.5 Pa, power of 50 W, and Ar flow rate of 40 sccm, respectively [43]. Thereafter, the Au film was deposited on the ZnO films by DC sputtering for 30 s at a power of 20 W. Next, the Au film was converted into separating Au NPs by annealing the samples at 500 °C for 1 h in a furnace under ambient N_2_. Finally, the prepared Au-ZnO film was treated by Ar plasma (CY-P2L-300W, CY Scientific Instrument Co., Ltd., Zhengzhou, China), which was operated at the working power and pressure of 100 W and 25 Pa, respectively. Here, treatment times of 0, 1, 3, and 5 min were indicated as S0, S1, S2, and S3, respectively. The experiment procedure process of Au-ZnO films with Ar plasma treatment is shown in Figure 13a [43]. Based on the SEM and XRD results, for untreated Au-ZnO film (S0) sample, it was observed that many Au NPs were distributed on the surface of Au-ZnO film due to annealing of Au layer in Figure 13b [43]. In addition, the size of Au NPs increases and the distribution becomes looser with increasing the plasma treatment time. Furthermore, the insets in the upper right corner of Figure 13b represent the size distribution of Au NPs. The average sizes of Au NPs on the surfaces of S0, S1, S2, and S3 are obtained to be 16.72, 17.77, 21.16, and 21.92 nm, respectively. The decreased density and the increased size of Au NPs would be attributed to recrystallization and growth due to the Ar plasma treatment, that is, the increase in Au NPs size occurs as its density becomes lower. 

**Figure 14:**
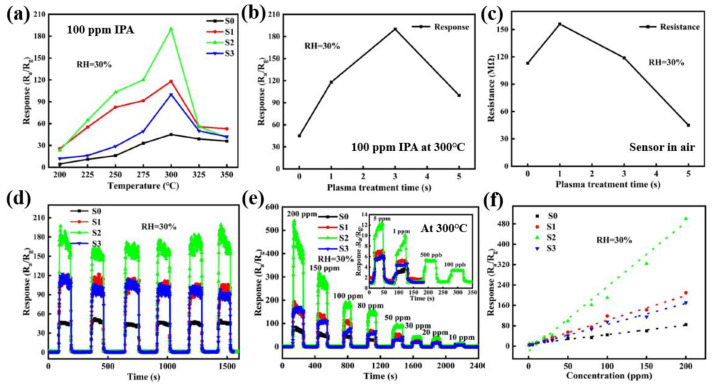
(**a**) Response of the four Au-ZnO sensors with different temperatures in the range from 200 to 350 °C at 100 ppm IPA. (**b**) Response of the sensors with different Ar plasma treatment time at conditions of 300 °C and 100 ppm IPA. (**c**) The change in the resistance of the four sensors in air with different Ar plasma treatment time. (**d**) Dynamic response properties of the four sensors for 6 cycles at conditions of 300 °C and 100 ppm IPA. (**e**) Dynamic response/recovery properties of the four sensors with IPA concentration at 300 °C. (**f**) Linear fitting curves on the response properties of sensors with various IPA concentration, by Wang et al. Reproduced with permission from ref. [43].

The responses (R_a_/R_g_) of the four Au-ZnO sensors were investigated with different temperatures in the range from 200 to 350 °C at 100 ppm IPA. As shown in Figure 14a, the responses of the four sensors increase with increasing the temperature and then decrease above 300 °C. This reason can be described as that when the sensor works at a relatively low temperature, the reactivity of IPA is low and not enough to fully react with the adsorbed O_2_ on the surface of Au-ZnO film, which leads to a low response value of sensor. Therefore, as the operating temperature of the four sensors increases, the reactivity of IPA increases and can fully react with O_2_ on the surface of ZnO, which will largely improve the response value. However, when the operating temperature is too high, the desorption rate of IPA would be larger than the adsorption rate, resulting in a decrease in the response with further increase in temperature. Thus, the sensor operating temperature condition is determined at 300 °C for maximum response. In particular, as shown in Figure 14b, the resistance of the sensors increased with increasing the plasma treatment time and then S2 sample has the highest response value for 100 ppm IPA at 300 °C. Moreover, Figure 14c shows the dependence of the resistance of the sensors in air on the Ar plasma treatment time. Furthermore, the dynamic response properties of the four sensors were investigated for 6 cycles at conditions of 300 and 100 ppm IPA. As shown in Figure 14d, it was confirmed that the four sensors operated well while maintaining their initial response characteristics for 6 cycles at conditions of 300 and 100 ppm IPA. Moreover, the response/recovery properties of the four sensors at 300 °C increased with increasing the IPA concentration, as shown in Figure 14e. Furthermore, the response of sensor depends on the IPA concentration according to the linear fitting curves, as shown in Figure 14f. 

**Figure 15:**
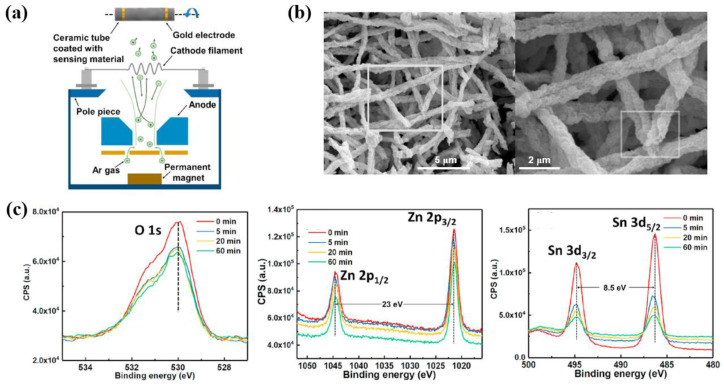
(**a**) Experimental setup of the plasma treatment process, by Hu et al. [44]. (**b**) SEM images and (**c**) the high-resolution XPS spectra on the O1s, Zn2p, and Sn3d peaks of ZnO–SnO_2_ nanofibers. treated by Ar plasma with different treatment time conditions. Reproduced with permission from ref. [44].

Hu et al. [44] investigated the response performance of gas sensor with ZnO–SnO_2_ NFs treated by plasma with different plasma treatment times. For plasma treatment, firstly, the ZnO–SnO_2_ NFs were prepared by electrospinning, and then were heated at 600 °C for 2 h in a furnace to remove the solvent. After that, as shown in Figure 15a, the prepared ZnO–SnO_2_ NFs were treated by Hall ion source using Argon (Ar) gas flow with various treatment time conditions, such as 0, 5, 20, and 60 min. For plasma formation, the cathode voltage, cathode current, anode voltage, and anode current were used as 14.2 V, 10.0 A, 150 V, and 1.9 A, respectively. To carry out uniform plasma treatment, the ceramic tube rotated during plasma treatment [44]. From the SEM images in Figure 15b, the morphology of ZnO–SnO_2_ NFs showed a thinner and continuous one, which was randomly stacked in layers without orientation. The width of the NFs was measured to be within a range from 200 to 500 nm [44]. Moreover, the element compositions of plasma–treated NFs were measured by X–ray photoelectron spectroscopy (XPS). As shown in Figure 15c, it was confirmed that the Zn and Sn elements in the NFs originated from compounds of ZnO and SnO_2_.

**Figure 16:**
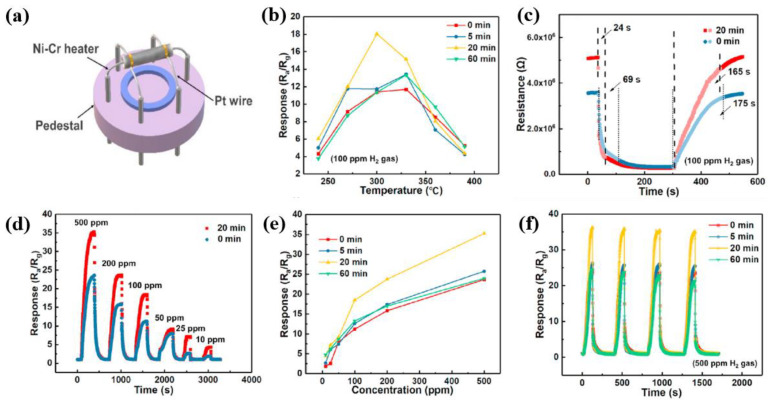
(**a**) Schematic diagram of heater-type gas sensor, by Hu et al. [44]. (**b**) The response properties of the four gas sensors with plasma-treated ZnO–SnO_2_ NFs at various operating temperatures under H_2_ gas flow of 100 ppm. (**c**) Response and recovery properties of the two gas sensors at optimum operating temperature. (**d**) Response property of the two gas sensors with increasing the different IPA concentration from 10 ppm to 500 ppm H_2_ gas at optimum operating temperature and (**e**) response property of the four gas sensors with plasma-treated ZnO–SnO_2_ NFs according to the different IPA concentrations. (**f**) The repeatable response of the four gas sensors with plasma-treated ZnO–SnO_2_ NFs with increasing the plasma treatment time in 500 ppm H_2_ gas. Reproduced with permission from ref. [44].

Figure 16a shows the schematic diagram of heater-type gas sensor [44]. In this sensor, there are two gold electrodes on the surface of ceramic tube, which are connected through Pt wire respectively. The Ni–Cr heater was penetrated through the center of ceramic tube, and six pins were attached on the pedestal for gas sensor measurement. The responses of the four gas sensors with plasma-treated ZnO–SnO_2_ NFs were measured with different operating temperatures at 100 ppm of H_2_ gas. As shown in Figure 16b, the response of the four gas sensors increased initially and then decreased with increasing the operating temperature over 300 °C. In particular, after plasma treatment for 20 min, the response property of gas sensors shows a maximum performance at 300 °C [44]. As shown in Figure 16c, the response and recovery time of gas sensors with plasma-treated NFs for 20 min were decreased compared to the non-treated NFs [44]. In Figure 16d, when compared to the untreated NFs sensor, the response of gas sensor with plasma-treated NFs for 20 min was increased with various H_2_ gas concentrations in range from 10 to 500 ppm [44]. Additionally, as shown in Figure 16e, the response of the four gas sensors was enhanced with increasing the gas concentration. Furthermore, the response repeatability of the four gas sensors was evaluated with different plasma treatment times in H_2_ gas flow of 500 ppm. Furthermore, the gas sensors of plasma-treated NFs for 20 min exhibited the higher response performance, as shown in Figure 16f [44]. On the contrary, the response of gas sensors with plasma-treated NFs for 60 min was decreased [44]. Table 3 summarizes the plasma surface modification of piezoelectric ZnO film discussed in this section.

#### 2.2.2. Plasma Surface Modification of Piezoelectric Polymer Using Plasma Process

**Figure 17:**
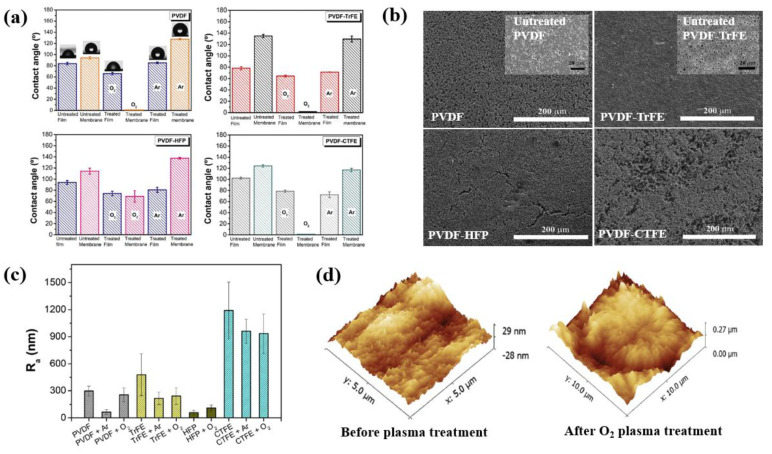
(**a**) Contact angle of PVDF and PVDF copolymer films and membranes with O_2_ and Ar plasma treatments over 600 s. (**b**) SEM images of all treated membranes (PVDF, P[VDF–TrFE], PVDF–HFP, and PVDF–CTFE) under O_2_ over 600 s. (**c**) Surface roughness for all plasma-treated samples and (**d**) 3D AFM images of PVDF and PVDF–HFP samples before and after O_2_ plasma treatments at conditions of 600 s and 100 W under O_2_ and Ar, by Correia et al. Reproduced with permission from ref. [45].

Correia et al. [45] investigated the surface properties of PVDF and its copolymers, including P[VDF–TrFE], poly (vinylidene fluoride–hexafluoropropylene) (PVDF–HFP), and poly(vinylidene fluoride-chlorotrifluoroethylene) (PVDF–CTFE) films, after APP surface treatment with various plasma treatments by using an O_2_ and Ar gas. Polymer surface modification was performed through plasma generated by a Zepto plasma chamber (Diener Electronic, Ebhausen, Germany) equipped with a 40 kHz RF plasma generator under a base pressure of 20 Pa. Ar and O_2_ gas were used to form the plasma and the polymer surface was treated at a plasma power of 100 W and total pressure of 80 Pa, with increasing treatment times from 200 to 600 s [45]. As displayed in Figure 17a, the contact angle of the plasma-treated polymer film and membrane surface decreased after plasma treatment, suggesting a transition to a more hydrophilic surface. From the SEM images in Figure 17b, it is evident that there was no significant change in surface morphology depending on plasma treatment. In addition, Figure 17c demonstrates that the surface roughness of the plasma-treated PVDF and its copolymers decreased compared to the non-treated polymer. However, the average surface roughness (R_a_) of the O_2_-treated samples was higher compared to the Ar-treated samples presented in Figure 17d [45].

**Figure 18:**
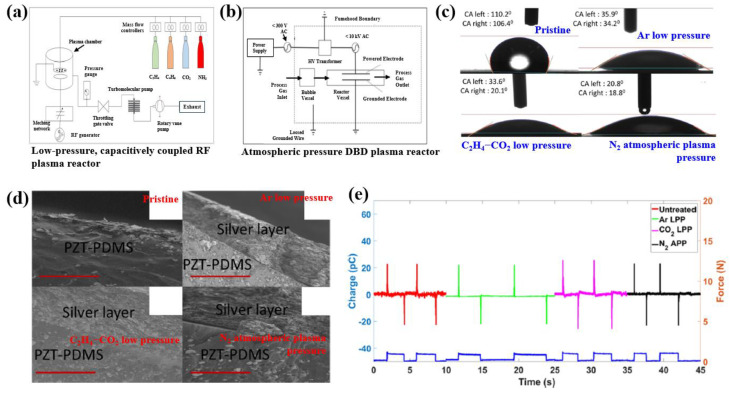
Schematic diagram of (**a**) LPP and (**b**) N_2_ APP surface treatment of PZT-PDMS films, by Sappati et al. [46]. (**c**) Water contact angle (WCA) of plasma-treated PZT-PDMS films under various LPP and N_2_ APP treatment conditions. (**d**) Cross-sectional SEM images of Ag layer printed on the plasma-treated PZT-PDMS films. (**e**) Piezoelectric charge properties of PZT-PDMS films before and after LPP and N_2_ APP treatments. Reproduced with permission from ref. [46].

Sappati et al. [46] investigated the use of low-pressure plasma (LPP) and APP to modify the surface of PZT-polydimethylosiloxane (PZT-PDMS) composite films for metallic silver (Ag) layer deposition. Before surface treatment, PZT NPs (28 vol %) were added to the PDMS solution, and the mixed solution was then stirred by hand for 15 min to fabricate the PZT-PDMS composite film. Subsequently, the PZT-PDMS solution was deposited using a spin-coating technique on FR-4 substrates at a speed of 1000 rpm for 15 s. After spin coating, the PZT-PDMS composite films were cured in an oven at 120 °C for 20 h, after which the PZT-PDMS composite films were peeled from the FR-4 substrates. To treat the surface of the PZT-PDMS films, two different plasma systems were used. As displayed in Figure 18a, low-pressure capacitively coupled RF glow discharge was used to conduct the plasma treatment on the surface of the PZT-PDMS films, under low-pressure C_2_H_4_-CO_2_ and Ar gas. The APP treatment was conducted under an N_2_ environment with a 300 V input voltage, a frequency of 21.5 kHz, and a treatment time of 2 min, as displayed in Figure 18b. As demonstrated in Figure 18c, the PZT-PDMS films were hydrophobic before plasma treatment, with a high water contact angle (WCA). Accordingly, the PZT-PDMS samples were treated by plasma with different LPP and N_2_ APP treatments to improve the wettability of PZT-PDMS surfaces. From the WCA results displayed in Figure 18c, it is evident that the contact angle of all plasma-treated PZT-PDMS composite films decreased after LPP and N_2_ APP treatment. Cross-sectional SEM images also indicated that the Ag layer was well maintained with strong adhesion on the plasma-treated PZT-PDMS films after the adhesion tests, as displayed in Figure 18d. In contrast, an Ag layer could not be observed on the untreated composite film. As depicted in Figure 18e, the piezoelectric charges of the untreated and plasma-treated composite film samples (Ar LPP, C_2_H_4_-CO_2_ LPP, and N_2_ LPP) were 2.7, 22, 25.7, and 23.1 pC for 1 N force, respectively.

**Figure 19:**
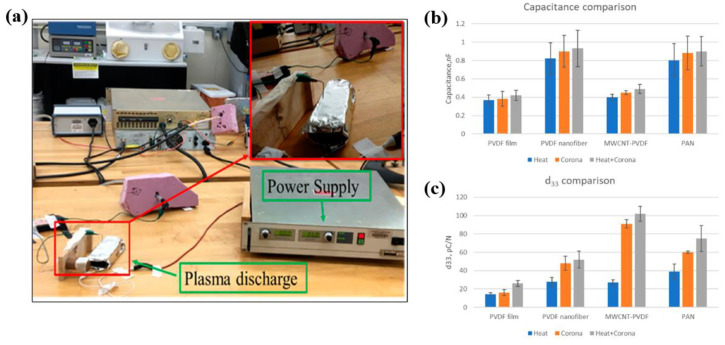
(**a**) Experimental set–up for APP corona discharge treatment, by Sultana et al. (**b**) Capacitance and (**c**) d_33_ of all nanofiber membranes and films. Reproduced with permission from ref. [47].

Sultana et al. [47] investigated the effect of APP corona discharge treatment on various piezoelectric polymer samples, including PVDF nanofibers, carbon nanotubes (CNT)–PVDF nanocomposites, and PAN nanofiber membranes. These samples were fabricated by electrospinning. To treat the piezoelectric polymers, plasma was produced using high-voltage power with a discharge current of 1 mA and output and discharge voltages of 6 kV, as indicated in Figure 19a. After plasma treatment, piezoelectric sensors were fabricated from the plasma-treated samples (including PVDF, MWCNT–PVDF, and PAN nanofibers) to evaluate the piezoelectric properties. As displayed in Figure 19b, the capacitances of all the plasma-treated films and nanofiber membranes were higher compared to the PVDF film. Moreover, the d_33_ values of all the plasma-treated samples increased, as indicated in Figure 19c. This increase in d_33_ was attributed to the increased capacitance caused by plasma treatment. 

**Figure 20:**
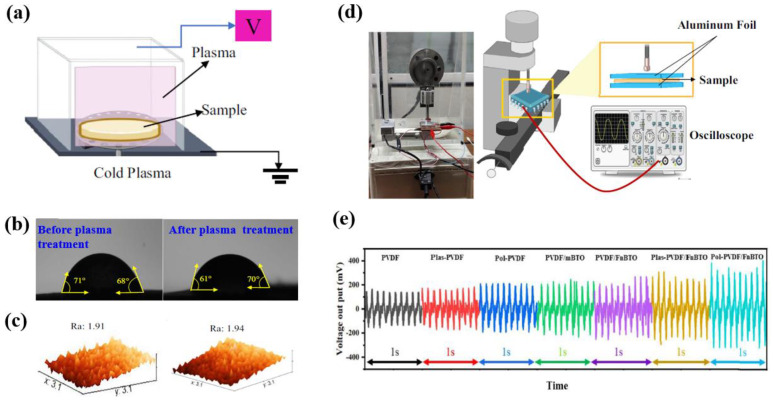
(**a**) Schematic diagram of experimental setup for plasma surface treatment of PVDF/BaTiO_3_ nanocomposite films. (**b**) WCA and (**c**) 3D AFM images of PVDF/BaTiO_3_ nanocomposite films before and after corona–plasma treatment. (**d**) Photograph and schematic diagram for piezoelectric measurements. (**e**) Piezoelectric output voltage of PVDF/BaTiO_3_ composite with different plasma-treated samples, by Fathollahzadeh et al. Reproduced with permission from ref. [48].

Fathollahzadeh et al. [48] investigated PVDF/BaTiO_3_ composites prepared by solution casting using nanoparticles and microparticles of BaTiO_3_. To increase the β-phase of the PVDF, piezoelectric BaTiO_3_ particles were incorporated within the PVDF polymer matrix. Subsequently, the PVDF/ BaTiO_3_ composites were modified by plasma treatment under inert helium (He) gas to improve the hydrophilic surface, as displayed in Figure 20a. As demonstrated in Figure 20b, the contact angle of the plasma–treated PVDF/BaTiO_3_ composites decreased from (71°–68°) to (61°–70°) due to changes in the surface properties caused by the plasma [48]. From the AFM results in Figure 20c, it is evident that the surface roughness of the PVDF/BaTiO_3_ nanocomposite films increased after plasma treatment. In addition, as depicted in Figure 20d, the piezoelectric output voltages of the PVDF/BaTiO_3_ composite films were obtained at a force of 2.6 N and a frequency of 5 Hz. As demonstrated in Figure 20e, the piezoelectric output voltage of the plasma–treated PVDF/BaTiO_3_ composites samples increased to 1.53 mV. Moreover, after incorporating functionalized BaTiO_3_ NPs within the PVDF polymer matrix, the piezoelectric output voltage increased compared to the sample with incorporated micron–sized BaTiO_3_.

**Figure 21:**
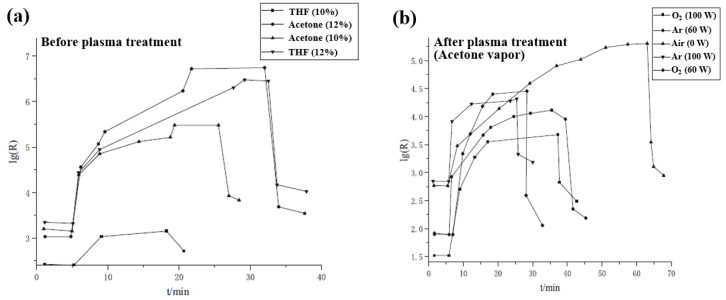
Piezoelectric response of PVDF/CB composite membrane with various vapor condition (**a**) before plasma surface treatment and (**b**) after plasma surface treatment to acetone vapor, by Wang et al. Reproduced with permission from ref. [49].

Wang et al. [49] investigated the effect of gas–sensitive response for plasma–treated PVDF/carbon black (CB) composite film. Figure 21a shows the piezoelectric response signals of the composite film with various vapor conditions of acetone and tetrahydrofuran (THF). In case of non–treated composite film, it reacted with a slow response rate to acetone THF vapor. Meanwhile, as displayed in Figure 21b, the gas response speed of PVDF/CB composite film was significantly improved after the composite film was treated by Ar and O_2_ plasma under atmospheric conditions, and the maximum piezoelectric response was reached in a very short time. This improvement in the gas response characteristics was attributed to the formation of a cross–linked layer on the PVDF/CB film, which was caused by the plasma treatment. Thus, the plasma–treated PVDF/CB composite film improved the ability of adsorption and desorption for gas molecules, resulting in the piezoelectric gas sensor device displaying good response performance [49]. Table 4 summarizes the plasma surface modification of piezoelectric polymers using plasma techniques presented in this section. 

#### 2.2.3. Plasma Surface Modification of Triboelectric Polymer Using Plasma Process

**Figure 22:**
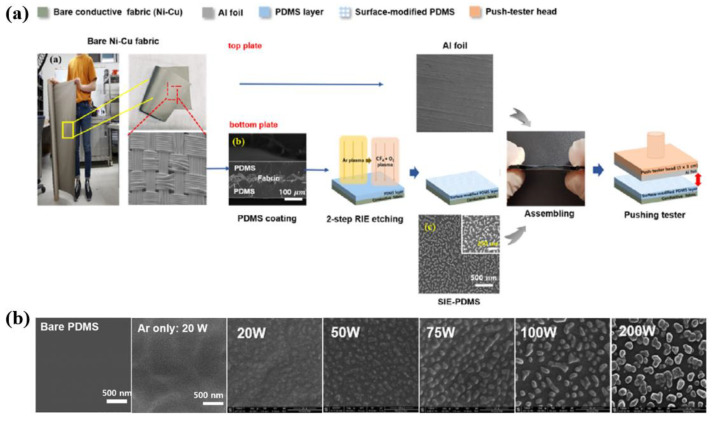
(**a**) Schematic diagram of the experimental procedure for T–TENG with nanostructure configurations on PDMS treated by using RIE plasma. (**b**) SEM images of plasma-treated PDMS with different RF power conditions, by Lee et al. Reproduced with permission from ref. [50].

Lee et al. [50] investigated enhancing the electrical performance of textile TENG (T–TENG) by using plasma-modified PDMS layers on conductive Ni-Cu textile substrates. As displayed in Figure 22a, the PDMS surface was treated with two-step reactive-ion etching (RIE) plasma with Ar and CF_4_ + O_2_ gas under a base pressure of 5 × 10^−5^ Torr. First, the PDMS was pre-treated using Ar plasma with an Ar gas flow rate of 40 sccm at a 20 W RF power for 10 min under a pressure of 10 mTorr [50]. After completing pretreatment, the second treatment was conducted using CF_4_ + O_2_ plasma with CF_4_ of 30 sccm and O_2_ gas of 10 sccm mixture gas to form the geometric configurations. From the SEM results in Figure 22b, it is evident that the surface of the plasma-treated PDMS was changed into nanostructural configurations with a high surface roughness under different RF power conditions [50]. 

**Figure 23:**
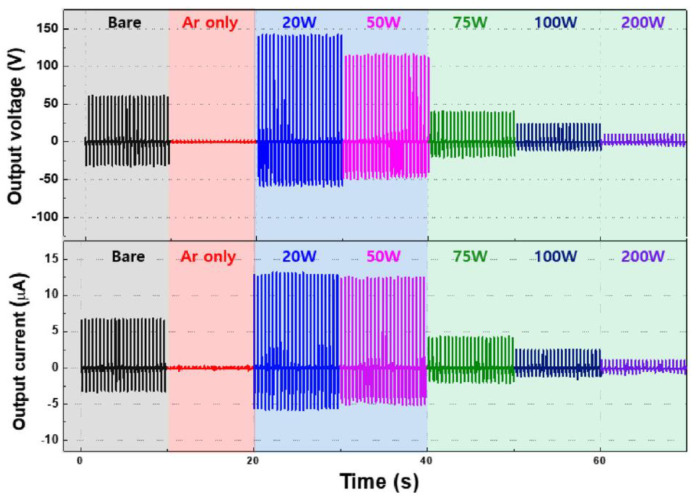
Output voltages and current obtained from T–TENGs with different plasma–treated PDMS according to various RF power conditions, by Lee et al. Reproduced with permission from ref. [50].

For two-step plasma treatment, the molecular bonds of the PDMS surface are first broken by Ar plasma. These broken bonds can strongly attract reactive species, such as F, CF_3_, CF_3_^+^, and O_2_^−^, which are dissociated and ionized by the mixed CF_4_ + O_2_ plasma in the second step, forming fluorocarbon (C–F) bonds on the PDMS surface. For this reason, the C–F bonds significantly affect the output performance of T–TENGs because of their higher electron affinities [50]. Therefore, after two-step Ar and CF_4_ + O_2_ plasma treatment, the maximum electrical output voltage and current generated in the T-TENGs was due to the presence of the F element on the plasma-treated PDMS with a nanostructure, since the formed F element has a strong electron affinity due to its large electronegativity F element, as indicated in Figure 23. 

**Figure 24:**
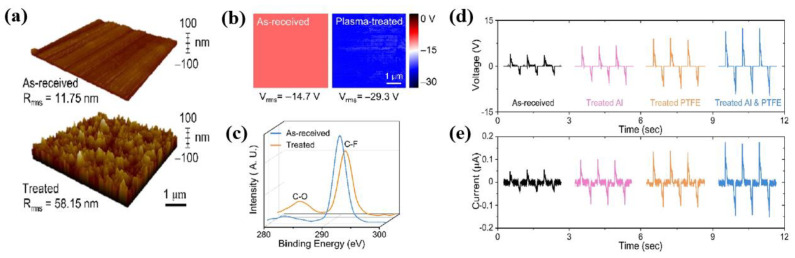
(**a**) Three-dimensional AFM images, (**b**) surface potential, and (**c**) XPS spectra of as-received and Ar plasma–treated PTFE samples. Comparison of (**d**) output voltage and (**e**) current of TENG device at 30 N before and after plasma surface treatments, Kong et al. [51].

Kong et al. [51] investigated the electrical characteristics (voltage and current) of a TENG device with a plasma-treated polytetrafluoroethylene (PTFE). The PTFE sample was treated with Ar plasma at 50 mW and Ar gas for 2 min. From the AFM results presented in Figure 24a, it is evident that the surface roughness of the PTFE increased after plasma treatment. In addition, the surface potential of the PTFE changed to a negative shift from +14.7 to −29.3 V, as indicated in Figure 24b. This negative shift improved the electrical performance of the TENG device due to the larger amounts of transfer charge carriers produced by the Ar plasma treatment [51]. Moreover, based on the X-ray photoelectron spectroscopic (XPS) spectra presented in Figure 24c, it is evident that the plasma-treated PTFE surface changed the chemical bonding of the PTFE surface. In other words, the peak of the C–O bond increased and the peak of the C–F bond decreased. As displayed in Figure 24d,e, the rotation-folding kirigami TENG device using plasma-treated PTFE produced a higher voltage (12.5 V) and current (176.8 nA) in the rotational mode compared to pristine PTFE (3.7 V and 57 nA, respectively) [51]. 

**Figure 25:**
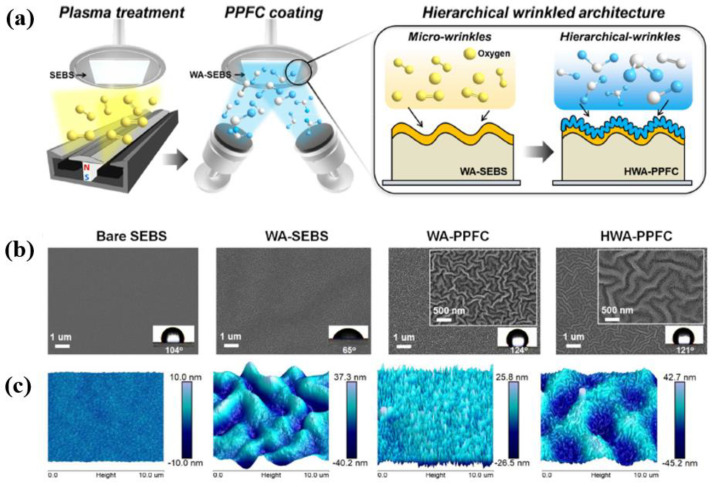
(**a**) Schematic diagram of HWA formation. (**b**) SEM and (**c**) 3D AFM images of SEBS, WA-SEBS, WA-PPFC, and HWA-PPFC samples, by Cho et al. Reproduced with permission from ref. [52].

To enhance the electrical performance of TENG devices, Cho et al. [52] proposed a hierarchical wrinkled architecture (HWA)–TENG that combined chemical surface modification (CSM) and physical surface modification (PSM). To produce the HWA–TENG with dual-wavelengths (microsize of 3.1 μm and nanosize of 311.8 nm), as displayed in Figure 25a, the transparent styreneethylene–butylene–styrene (SEBS) substrates were treated by a linear ion source (LIS, LIS450, Advanced thin film). First, a SEBS liquid solution was prepared by dissolving SEBS powder in toluene (weight ratio, 4:10). Next, the SEBS film was formed on PET with a thickness of 20 μm and then dried at 60 °C for 10 min. The formed SEBS film was then treated with plasma using a direct current power supply (Forte I–302, EN Technologies Inc., Gunpo-si, Gyeonggi-do, Republic of Korea) at 10 W for 1 min. To produce the plasma, Ar, O_2_, and nitrogen (N_2_) gases were provided at the same flow rate of 60 sccm [52]. The SEM results displayed in Figure 25b confirm that the pristine SEBS surface was flat and smooth. In contrast, the SEBS surface treated by O_2_ plasma changed into a microscale-wrinkled architecture (WA--SEBS). The WA-SEBS treated by O_2_ and N_2_ plasma formed wrinkles with a larger amplitude and wavelength compared to WA-SEBS treated by Ar. The AFM images of WA–SEBS in Figure 25c indicate that the amplitudes of the wrinkles were 26.5 nm (Ar), 43.5 nm (O_2_), and 40.5 nm (N_2_), depending on the process gas. In addition, the wavelengths of the wrinkles had microscales of 1.8 μm, 3.0 μm, and 3.1 μm, respectively. After completing plasma treatment, the plasma polymer–fluorocarbon (PPFC) thin film was deposited on the HWA surface by a sputtering method using a CNTs–PTFE composite target. The wrinkle dimensions of the HWA–PPFC were smaller than that of WA-SEBS and larger than that of WA–PPFC. Finally, compared to WA–PPFC, the HWA–PPFC surface had nano–wrinkles with a wider wavelength through PPFC film deposition and surface modification by O_2_ plasma.

**Figure 26:**
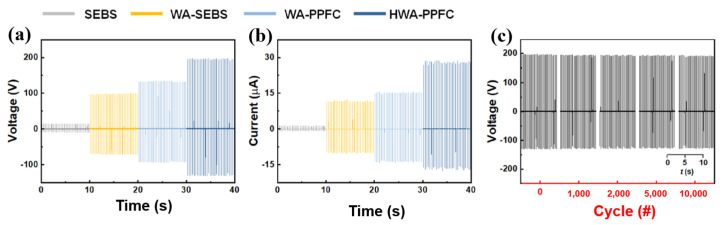
Output (**a**) voltage and (**b**) current of TENG devices with different samples. (**c**) Mechanical stability of HWA–TENG device under bending motions during 10,000 cycles, by Cho et al. Reproduced with permission from ref. [52].

Based on the TENG results presented in Figure 26a,b, the output voltage and current signals of TENG devices with four friction layers were investigated under contact motion under the conditions of a force of 30 N and a frequency of 3 Hz. For the SEBS sample, the electrical output signal of the TENG device was not observed. In the case of the HWA–PPFC, the electrical output and current significantly increased. The output and current of the TENG devices applied with HWA–PPFC were 200 V and 30 μA, respectively [52]. This improvement in electrical properties was attributed to the changes in surface properties, such as the surface contact and electronegativity due to the micro-wrinkle structure caused by O_2_ plasma. This enhanced the device’s ability to attract negative charges through the PPFC thin film with a high surface potential. Furthermore, to evaluate the feasibility of HWA–TENGs in wearable applications, the bending stability was assessed during 10,000 cycles at a frequency of 1 Hz with an HWA surface. As displayed in Figure 26c, the output voltage of the TENG device was accurately maintained during 10,000 cycles, indicating high mechanical stability. 

**Figure 27:**
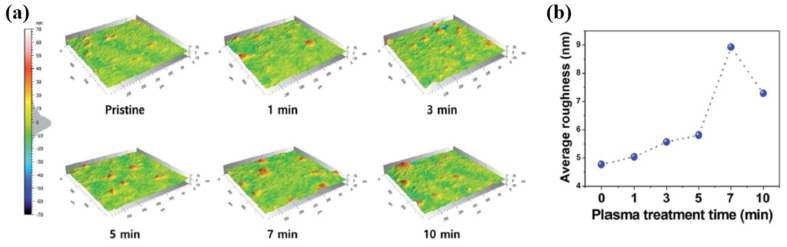
(**a**) Three-dimensional confocal microscope images and (**b**) average surface roughness values of plasma-treated PDMS with different treatment times at a plasma power of 100 W, by Lee et al. Reproduced with permission from ref. [53].

Lee et al. [53] investigated a high-performance TENG device fabricated with PDMS composite film that contained surface-modified carbon nanotubes (SMCs). To fabricate the SMC–PDMS composite film, the PDMS was prepared using a mixture of a base resin and a curing agent with a weight ratio of 10:1. To prepare the PDMS composite films, the SMCs were initially dispersed in toluene and then mixed with an elastomer. Then, the mixed solution was stirred until the SMCs were completely dissolved. After degassing under a vacuum for approximately 30 min, the mixed PDMS solution was then poured into a petri dish and cured for 1 h. The formed SMC–PDMS composite films were then treated by plasma produced with an RF power of 100 W, a pressure of 10 mTorr, and gas conditions of CF_4_ (40 sccm) and O_2_ (10 sccm) [53]. Based on the experimental results obtained using a confocal microscope, as presented in Figure 27a,b, the R_a_ of PDMS increased with increasing treatment time. The maximum R_a_ was obtained in the PDMS treated by RF plasma for 7 min. Moreover, as displayed in Figure 27b, after further plasma treatment (7 min), the R_a_ of the PDMS decreased. This reduction in roughness was considered to be due to the surface damage caused by plasma treatment for more than 7 min. 

**Figure 28:**
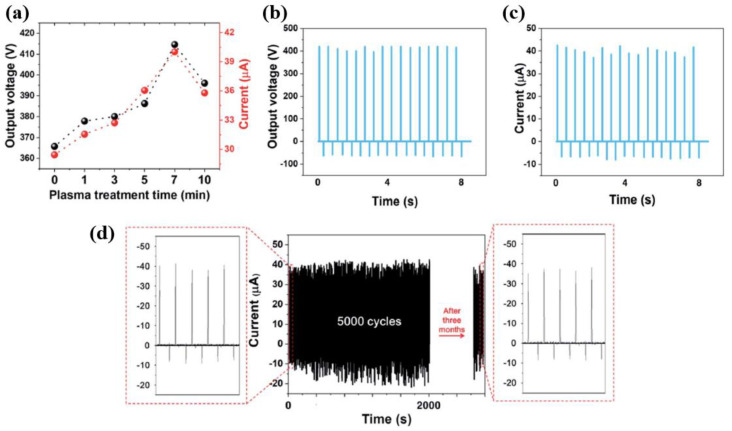
(**a**) Electrical output voltage and current of TENG device with plasma-treated PDMS under various treatment times at a power of 100 W, by Lee et al. [53]. (**b**) Output voltage and (**c**) current signals of TENG device with optimized SMC–PDMS in the contact–separation mode. (**d**) Stability and durability results of TENG device with optimized SMC–PDMS during 5000 cycles and after 3 months. Reproduced with permission from ref. [53].

As demonstrated in Figure 28a, the electrical output voltage and current of the TENG device with a plasma-treated PDMS increased with increasing treatment times. In addition, the maximum values of output voltage and current of the TENG device were obtained in the SMC–PDMS composite films treated by RF plasma for 7 min. These maximum electrical parameters of the TENG device were related to the high surface roughness induced by RF plasma. As depicted in Figure 28b,c, the output voltage and current were 414.63 V and 40.03 μA, respectively. These results represented increases of 184% and 330% compared to the values for the TENG device with pristine PDMS, respectively. The SMC–PDMS sample treated by plasma for 7 min was then placed for 3 months under an ambient atmosphere. The performance of the device was then measured to evaluate its stability. As displayed in Figure 28d, the output performance of the TENG device with a plasma-treated PDMS decreased due to fluorine losses on the surface of the PDMS. Nevertheless, the plasma-treated SMC–PDMS maintained the TENG device’s output performance successfully.

**Figure 29:**
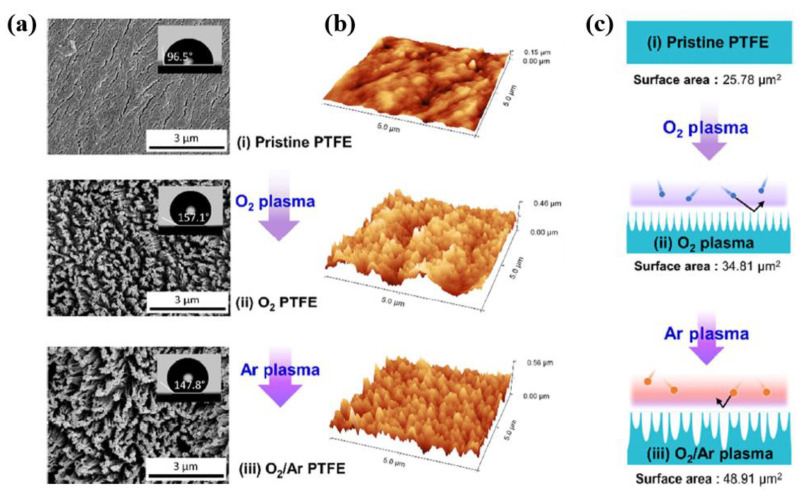
(**a**) SEM with contact angles and (**b**) AFM images of PTFE surface before and after two-step plasma using O_2_ and O_2_/Ar gas. (**c**) Schematic diagram of two-step O_2_/Ar plasma with various samples (pristine, O_2_, and O_2_/Ar plasma-treated PTFE surfaces), by Prada et al. Reproduced with permission from ref. [54].

Prada et al. [54] investigated modifying the surface of PTFE using O_2_/Ar plasma etching to enhance the triboelectrification efficiency of TENG devices. The PTFE surface was treated by plasma using a capacitively coupled plasma (CCP) reactor. Parallel plate-shaped electrodes with a diameter of 19 cm were used as the powered and ground electrodes, and the gap between the two electrodes was set to 12 cm. The bottom electrode was connected to a bipolar pulse power supply (AE Pinnacle PLUS+, Advanced Energy) and the top electrode was connected to ground. The PTFE samples were placed on the bottom electrode [54]. Plasma discharge was produced using a power of 100 W, a frequency of 50 kHz, and O_2_ and Ar gas at a flow rate of 10 sccm under a background pressure of 4 × 10^−3^ Pa in a vacuum system [54]. As displayed in Figure 29a,b, the SEM images with a WCA, and 3D AFM images of PTFE surface increased after plasma treatment and the plasma-treated PTFE surface was changed into a nanostructure with a high surface roughness [54]. The PTFE surface treated by one-step O_2_ plasma (O_2_, Ar/O_2_, and O_2_/Ar) changed into a surface shape with a high R_rms_ of 75.06 nm. This highest R_rms_ contributed to achieving the highest contact angle. In contrast, the PTFE surface treated by two-step O_2_/Ar plasma exhibited a slightly lower R_rms_ of 72.73 nm with a surface area of 48.91 μm^2^. This result indicated that the two-step O_2_/Ar plasma process formed fine nanostructures on the PTFE surface. Figure 29c presents a schematic diagram of the surface modification of PTFE by the plasma process (using O_2_/Ar) [54]. 

**Figure 30:**
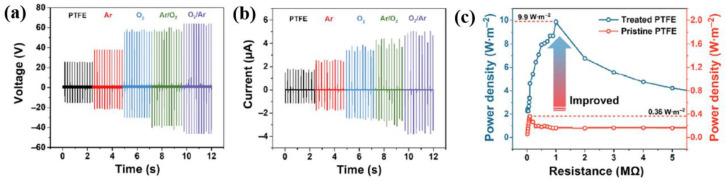
Electrical (**a**) output voltage, (**b**) current, and (**c**) power density properties of TENG devices with various samples (pristine PTFE, O_2_ plasma, and O_2_/Ar plasma), by Prada et al. Reproduced with permission from ref. [54].

The voltage and current properties of TENG devices with plasma-treated PTFE were evaluated at a frequency of 5 Hz under an applied mechanical force of 1 N [54]. As displayed in Figure 30a,b, the highest maximum output voltage and current values of PTFE treated by O_2_/Ar plasma were 110.3 V and 8.8 μA, respectively, which were three times larger than the values for the pristine PTFE-based-TENG devices [54]. The reason for this increase in voltage and current was the increased surface area due to the plasma treatment. In addition, as depicted in Figure 30c, the maximum power density of the TENG device with PTFE treated by O_2_/Ar plasma was 9.9 W/m^2^ at a load resistance of 1 MΩ, which was 27.5 times higher than that of the TENG devices with pristine PTFE [54]. This increase in the electrical properties of the TENG device was attributed to the defective bonds produced by plasma treatment acting as charge trapping levels, which was dependent on the triboelectric charge density [54].

**Figure 31:**
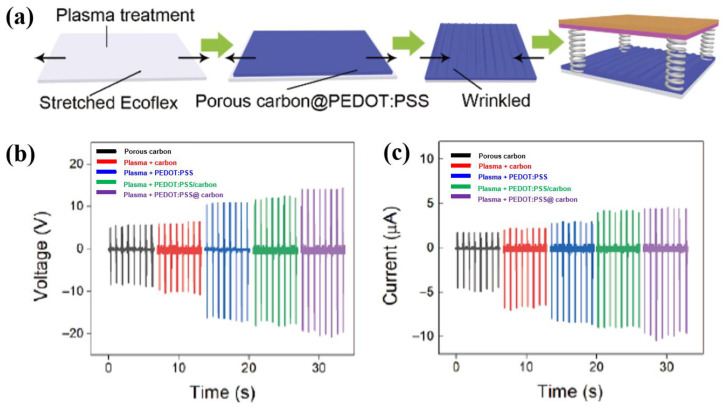
(**a**) Schematic diagram of experimental procedure for TENG fabrication. (**b**) Output voltage and (**c**) current of TENG devices with various plasma-treated electrodes, by Chen et al. Reproduced with permission from ref. [55].

Chen et al. [55] investigated the use of plasma-treated Ecoflex film to fabricate a poly(3,4-ethylenedioxythiophene):poly(styrenesulfonate) (PEDOT:PSS)/porous carbon electrode in a flexible TENG device. As depicted in Figure 31a, the porous carbon was prepared using candle soot powder, and then the porous carbon was dispersed into a PEDOT:PSS solution to make mixed solutions with various concentrations. The Ecoflex solution was deposited using a spin-coating technique on a silicon wafer and then cured at 100 °C for 2 h. The Ecoflex film was formed with a thickness of 0.3 mm, and was then treated by a plasma cleaner (Harrick plasma, PDC–002) [55]. This plasma surface treatment enabled substrate surface activation and contributed to improving surface adhesion due to the hydrophilic characteristics. Thus, the PEDOT:PSS/porous carbon was well deposited with strong adhesion through spin-coating on the plasma-treated Ecoflex film, which decreased the electrical resistance of the electrode. As displayed in Figure 31b, the TENG device was fabricated with a size of 1.5 cm^2^, and the measurements were performed at 80 N and a frequency of 1 Hz. The output voltages of the TENG devices under different conditions (porous carbon, PEDOT:PSS, PEDOT:PSS/porous carbon, and plasma-treated porous carbon@PEDOT:PSS) were 10.2, 16.5, 17.6, and 19.9 V, respectively. In addition, the corresponding output currents were 4.7, 6.7, 8.3, 8.9, and 9.8 μA, respectively, as displayed in Figure 31c [55]. 

**Figure 32:**
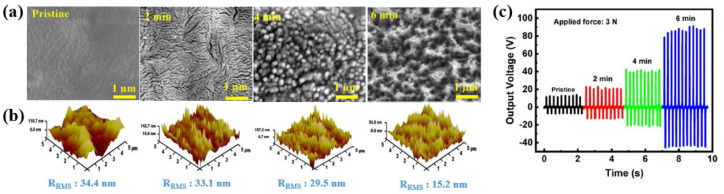
(**a**) SEM and (**b**) 3D AFM images of PTFE before and after plasma treatment with increasing treatment time. (**c**) Output voltages of TENG devices with pristine and various plasma-treated PTFEs according to different treatment times, by Ahmed et al. Reproduced with permission from ref. [56].

Ahmed et al. [56] investigated the enhancement of TENG efficiency through surface modifications of PTFE using low-pressure air plasma. The PTFE samples were placed in a vacuum chamber and air plasma was produced at an RF power of 60 W under low pressure (~1 × 10 mbar) conditions. The PTFE samples were treated using low-pressure air plasma with different treatment times (2, 4, and 6 min) [56]. The surface morphologies of the PTFE before and after plasma treatment were evaluated using SEM and AFM analyses. As depicted in Figure 32a,b, the pristine PTFE had a wave surface with a valley-like structure, meaning its root-mean-square surface roughness (R_RMS_) was high (34.4 nm). After plasma treatment for 2 min, the PTFE surface changed to a nanotextured structure surface. When the treatment time was increased to 4 and 6 min, the R_RMS_ values were reduced to 29.5 and 15.2 nm, which was confirmed by SEM and AFM. These results inferred that the decrease in R_RMS_ caused the formation of a uniform and nanotextured surface. As a result, as displayed in Figure 32c, the output voltage of the TENG devices with plasma-treated PTFE increased from 13 to 90 V under an applied force of 3 N compared to the untreated PTFE. In particular, the highest electrical power density of a TENG device with plasma-treated PTFE (6 min) was 3.2 W/m^2^, which was higher than that of the pristine PTFE (0.133 W/m^2^). The improvement in efficiency of the TENG devices with plasma-treated PTFE was attributed to the formation of a nanostructured morphology and chemical modification of the PTFE treated by plasma. Therefore, the nanostructured surface provided a higher contact area between the Al and PTFE. Additionally, C dangling bonds and new functional groups were formed on the PTFE surface, which acted as electron acceptor sites, improving the efficient transfer of surface charge electrons from the Al to PTFE [56]. 

**Figure 33:**
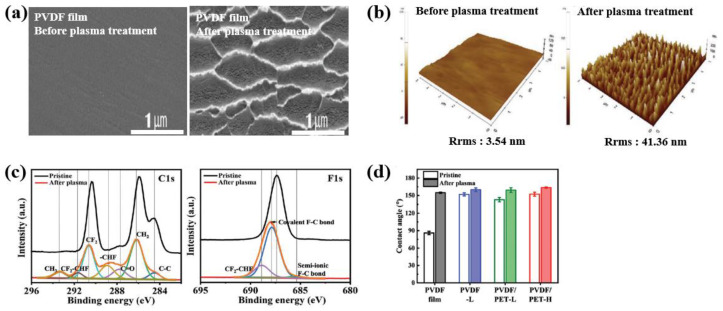
(**a**) FE–SEM, (**b**) 3D AFM images, (**c**) C1s and F1s curve-fitting results of XPS spectra, and (**d**) contact angle of PVDF film before and after plasma treatment with O_2_ plasma for 12 min and CF_4_ plasma for 4 min, by Hong et al. Reproduced with permission from ref. [57].

Hong et al. [57] investigated PVDF fabric samples subjected to plasma treatments using O_2_ and CF_4_ to improve the electrical performance of TENG devices with PVDF fabric [57]. All the PVDF fabric samples were treated by using RIE plasma chamber (Plasmalab 80Plus, Oxford Instrument PLC, Abingdon Oxon, UK) with O_2_ plasma for 12 min and CF_4_ plasma for 4 min at a power of 180 W under a pressure of 40 mTorr [57]. From the SEM and AFM results displayed in Figure 33a,b, it is evident that the R_RMS_ values of the plasma-treated PVDF samples increased and their surfaces changed to nanostructures with a reduced roughness. Figure 33c displays the carbon and fluorine curve-fitting results of XPS spectra for a plasma-treated PVDF surface. After plasma treatment, new peaks related to –CHF, CF_2_–CHF, and CF_3_–CH_x_ were observed on the plasma-treated PVDF surface. The increased fluorine content of the plasma-treated PVDF surface was attributed to the fluorine radicals generated by CF_4_ plasma treatment, which produced C–H and C–F covalent bonds on the PVDF surface [57]. In addition, as depicted in Figure 33d, the plasma treatment increased the contact angle [57]. 

**Figure 34:**
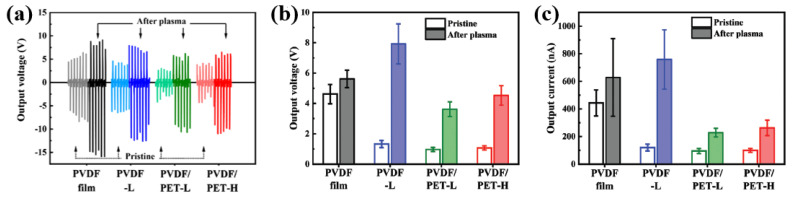
(**a**) Output voltage signals of TENG devices with various plasma-treated PVDF samples before and after plasma treatment with O_2_ plasma for 12 min and CF_4_ plasma for 4 min. Comparison of the (**b**) output voltages, and (**c**) currents of TENG devices with different plasma-treated samples before and after plasma surface treatment, by Hong et al. Reproduced with permission from ref. [57].

As depicted in Figure 34a, for all the plasma-treated PVDF samples, the TENG devices achieved higher output voltage and current values compared to the pristine PVDF sample. In addition, as displayed in Figure 34b,c, all the plasma-treated PVDF samples exhibited improved output voltage and current properties compared with the pristine PVDF sample. In particular, the plasma-treated PVDF fabric had a voltage output stability of 45% and a current output stability of 77%, resulting in the highest energy harvesting performance stability among the tested samples [57]. 

**Figure 35:**
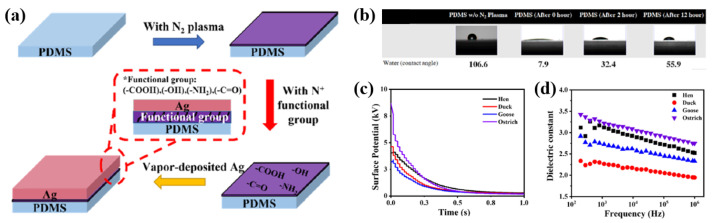
(**a**) Schematic diagram of experimental procedure of stretchable PDMS electrode, by Lin et al. (**b**) Contact angle results of PDMS substrates after plasma treatment with different plasma treatment times. (**c**) Surface potentials and (**d**) dielectric constants for various types of EMs. Reproduced with permission from ref. [58].

Lin et al. [58] demonstrated a simple and cost-effective method for fabricating TENG devices using eggshell membranes (EMs). Before coating a metallic electrode, the PDMS was treated by using a commercial N_2_ atmospheric plasma treatment (Harrick Plasma, PDC–32G) for 0, 2, and 12 h under an atmospheric environment to form a hydrophilic PDMS surface. As depicted in Figure 35a, the plasma power and N_2_ gas pressure were 18 W and 1.5 kgf/cm^2^, respectively [58]. As depicted in Figure 35b, the contact angle decreased from 106.6° to 7.9° after N_2_ atmospheric plasma treatment compared to the untreated PDMS, indicating that the PDMS surface was hydrophilic. In contrast, the contact angle increased from 7.9 to 55.9° after N_2_ atmospheric plasma treatment for 12 h [50]. Thereafter, the Ag layer was well deposited on the plasma-treated PDMS surface through vapor deposition. The thickness and sheet resistance of the Ag layer were approximately 50 nm and 1.34 Ω, respectively [58]. Figure 35c displays the amounts of charge transferred for the various types of EMs. Among these, the ostrich EM had the highest amount of transferred charge due to its good contact area and surface roughness. In addition, the dielectric constant of the ostrich EM was higher than the other samples due to the lower volume of pores and the higher amount of transferred charge, as displayed in Figure 35d [58].

**Figure 36:**
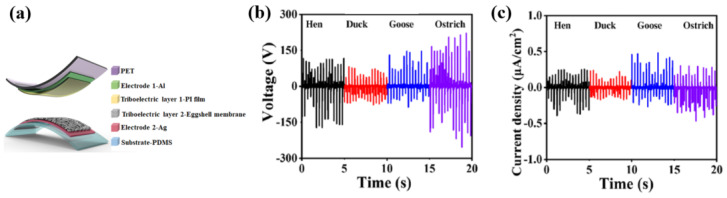
(**a**) Schematic diagram of EM-TENG device, by Lin et al. (**b**) Output voltages and (**c**) current density of ostrich EM-TENG device measured at 30 N and 5 kHz. Reproduced with permission from ref. [58].

Figure 36a represents the schematic diagram of EM-TENG device with a rectangular shape at a size of 2.0 × 2.0 cm^2^. One side of EM layer was used as the positive tribomaterial, and the other was attached to an Ag layer as an electrode. As for a counter-electrode, the polyimide (PI) film was used as the negative tribomaterial with aluminum (Al) tape with conductive Al tape covering the PI surface as the electrode. Figure 36b displays the maximum output voltage of TENG devices with various EMs under a cycled compressive force of 30 N at an applied frequency of 3 Hz. Under the same mechanical force, the output voltages of the hen, duck, goose, and ostrich EMs were approximately 250, 150, 200, and 300 V, respectively. In addition, the output current density of the ostrich EM was up to approximately 0.6 μA/cm^2^ higher than the values for the duck and goose EMs, as depicted in Figure 36c. Moreover, the resulting power of the TENG device with ostrich EM was 18 mW. This device also displayed good durability when subjected to 9000 cycles at 30 N at a frequency of 3 Hz [58]. 

Min et al. [59] demonstrated a new self-powered temperature sensor based on flexible TENGs. First, the performance of the TENGs was optimized using plasma treatment. For this, the PTFE film was treated by RIE plasma (RF 150 W power) using Ar gas to produce nanopatterns for a higher contact area between the interfaces. For the plasma, power was applied at 150 W (10 mL/cc) for durations of 3 to 12 min [59]. As a result, the pristine PTFE was flat and smooth [59]. In contrast, after plasma treatment, the plasma-treated PTFE surface exhibited a nanostructure pattern with a high degree of roughness. In addition, the transferred charges of the plasma-treated PTFE surface increased with increasing plasma treatment time [59]. Furthermore, the output voltages of the TENG devices with pristine and all plasma-treated PTFE increased from 9 V (pristine, 0 min) to 37 V (3 min), to 61 V (6 min), to 82 V (9 min), and to 92 V (12 min) [59]. In other words, there was a 10.2 times enhancement from the pristine PTFE to the 12 min plasma-treated PTFE. Thereby, the current density was approximately 0.12 μA/cm^2^ for the pristine (0 min) sample, and a maximum of 1.14 μA/cm^2^ (12 min) for the plasma-treated PTFE, representing a 9.2 times increase [59]. This maximum output current density was attributed to the increase in transferred charge due to plasma surface modification. Table 5 summarizes this section for plasma surface modification of triboelectric polymers using plasma techniques. 

## 3. Conclusions 

This review introduces recently developed plasma-based approaches for depositing and treating piezoelectric NPs and piezoelectric polymer films for NG and sensor applications. One approach is a plasma surface modification that can improve the surface charge density characteristics of piezoelectric polymers due to nanostructure formation and new functional groups on the polymer surface for improving the electrical performance of NG devices. The other is a plasma synthesis for piezoelectric materials under vacuum, low pressure, and ambient air conditions, which would highlight the existing challenges and future directions of plasma methods. As a result, the various plasma processes involved in piezoelectric NGs and sensors, including plasma-based vapor deposition, dielectric barrier discharge, and surface modification, are introduced and summarized for controlling various surface properties (etching, roughening, crosslinking, functionalization, and crystallinity).

## Figures and Tables

**Table 1:** Summary of the plasma deposition and synthesis process of ZnO NPs.

No	Object	Plasma Source	Application	Year	Author Reference
1	ZnO thin films	PECVD (2.45 GHz microwave, 500 W)	PENGs	2023	García–Casas et al. [35]
2	ZnO/CF composites	Plasma–liquid technology (1.5 kV)	PENGs	2023	Zhong et al. [36]
3	ZnO NPs	APPJ	PENGs	2023	Schwan et al. [37]
4	ZnO thin film	PE–ALD	PENGs	2020	Ali et al. [38]

**Table 2:** Summary of plasma deposition and synthesis process of piezoelectric polymers using APP technique.

No	Object	Precursor	Plasma Source	Application	Year	Author Reference
1	P[VDF-TrFE] copolymer thin film	5% P[VDF-TrFE] copolymer nano powder + 95% DMF solvent	Bipolar pulse (12.5 kV_p-p_, 26 kHz)	PENGs	2023	Jung et al. [39]
2	PVDF film	5% PVDF nano powder + 95% DMF solvent	Bipolar pulse (12.5 kV_p-p_, 26 kHz)	PENGs	2019	Jung et al. [40]
3	PVDF film	5% PVDF nano powder + 95% DMF solvent	Bipolar pulse (10 kV_p-p_, 26 kHz)	PENGs	2022	Bae et al. [41]

**Table 3:** Summary of plasma surface modification of the piezoelectric ZnO film for sensor application.

No	Object	Plasma Source	Application	Year	Author Reference
1	ZnO NFs	O_2_ and H_2_ plasma by ICPS (13.56 MHz, 30 Pa, 450 W)	Sensor	2020	Du et al. [42]
2	Au-ZnO films	Ar plasma (CY-P2L-300W) (25 Pa, 100 W)	Sensor	2023	Wang et al. [43]
3	ZnO–SnO_2_ NFs	Ar plasma by Hall ion source	Sensor	2020	Hu et al. [44]

**Table 4:** Summary of plasma surface modification of the piezoelectric polymer using plasma process.

No	Object	Plasma Source	Application	Year	Author Reference
1	PVDF and its copolymer (PVDF–HFP, P[VDF–TrFE], PVDF–CTFE)	RF frequency pulse (100 W, 40 kHz)	PENGs	2019	Correia et al. [45]
2	PZT-PDMS composite film	Low (or vacuum) and atmospheric pressure N_2_ plasma (300 V, 21.5 kHz)	PENGs	2021	Sappati et al. [46]
3	PVDF film and nanofiber	Atmospheric pressure corona plasma (6 kV)	PENGs	2023	Sultana et al. [47]
4	PVDF–BaTiO_3_ film	Atmospheric pressure plasma (12 kV, 5 kHz)	PENGs	2024	Fathollahzadeh et al. [48]
5	PVDF/CB composite film	Capacitively coupled plasma (13.56 MHz)	Sensor	2022	Wang et al. [49]

**Table 5:** Summary of plasma surface modification of triboelectric polymers using plasma techniques.

No	Object	Plasma Source	Application	Year	Author Reference
1	PDMS	RIE plasma (20 W)	TENGs	2019	Lee et al. [50]
2	PTFE	Ar plasma (50 mW)	TENGs	2021	Kong et al. [51]
3	SEBS	O_2_ plasma	TENGs	2022	Cho et al. [52]
4	SMCs–PDMS film	RF plasma (100 W)	TENGs	2022	Lee et al. [53]
5	PTFE	CCP plasma Ar and O_2_ plasma (100 W, 50 kHz)	TENGs	2022	Prada et al. [54]
6	Ecoflex film	Plasma cleaner (Harrick plasma, PDC-002)	TENGs	2022	Chen et al. [55]
7	PTFE	RF plasma (60 W)	TENGs	2023	Ahmed et al. [56]
8	PVDF fabrics	RIE plasma (Plasmalab 80Plus, UK) O_2_ and CF_4_ plasma (180 W)	TENGs	2023	Hong et al. [57]
9	Eggshell membranes (EMs)	N_2_ plasma (Harrick Plasma, PDC–32G) (18 W)	TENGs	2023	Lin et al. [58]
10	PTFE	Ar plasma (150 W)	TENGs	2023	Min et al. [59]

## Data Availability

Not applicable.

## Abbreviations

NPs	Nanoparticles
NGs	Nanogenerators
PENGs	Piezoelectric NGs
TENGs	Triboelectric NGs
ZnO	Zinc oxide
PZT	Lead zirconate titanate
BaTiO_3_	Barium titanate
PMN–PT	Lead magnesium niobate–lead titanate
PVDF	Polyvinylidene fluoride
P[VDF–TrFE]	Poly(vinylidenefluoride–co–trifluoroethylene)
PAN	Polyacrylonitrile
APP	Atmospheric pressure plasma
PECVD	Plasma-enhanced chemical vapor deposition
PMMA	Polymethylmethacrylate
SEM	Scanning electron microscope
CF	Carbon fiber
PFM	Piezoresponse force microscopy
d_33_	Piezoelectric coefficient
APPJ	Atmospheric pressure plasma jet
PE–ALD	Plasma-enhanced atomic layer deposition
PET	Poly(ethylene terephthalate)
ICCD	Intensified charge-coupled device
DMF	Dimethylformamide
FT-IR	Fourier transform infrared spectroscopy
Modified–APPDS	Modified APP deposition system
NFs	Nanofibers
RF	Radio frequency
ICPS	Inductively coupled plasma source
O_2_	Oxygen
H_2_	Hydrogen
Ar	Argon
XPS	X-ray photoelectron spectroscopy
PVDF–HFP	Poly (vinylidene fluoride–hexafluoropropylene)
PVDF–CTFE	Poly(vinylidene fluoride–chlorotrifluoroethylene)
R_a_	Average surface roughness
LPP	Low-pressure plasma
PDMS	Polydimethylosiloxane
PTFE	Polytetrafluoroethylene
Ag	Silver
WCA	Water contact angle
CNT	Carbon nanotubes
He	Helium
CB	Carbon black
THF	Tetrahydrofuran
T–TENG	Textile TENG
RIE	Reactive-ion etching
C–F	Fluorocarbon
HWA	Hierarchical wrinkled architecture
CSM	Chemical surface modification
PSM	Physical surface modification
SEBS	Styreneethylene–butylene–styrene
LIS	Linear ion source
PPFC	Plasma polymer–fluorocarbon
N_2_	Nitrogen
WA	Wrinkled architecture
PPFC	Plasma polymer–fluorocarbon
SMCs	Surface-modified carbon nanotubes
CCP	Capacitively coupled plasma
PEDOT:PSS	Poly(3,4-ethylenedioxythiophene):poly(styrenesulfonate)
R_RMS_	Root–mean–square surface roughness
Ems	Eggshell membranes
Al	Aluminum
PI	Polyimide
